# 453. Shorter Duration of Antimicrobial Therapy is Noninferior for Cardiovascular Implantable Electronic Device Associated Systemic Infections

**DOI:** 10.1093/ofid/ofaf695.152

**Published:** 2026-01-11

**Authors:** Emily Y Xiao, Patrick Lynch, Sarwat Khalil, Derrick Draeger, Alexandra Lewis, Faiz Baqai, Mihail Chelu, Muhammad Rizwan Sohail

**Affiliations:** Baylor College of Medicine, Houston, TX; Baylor College of Medicine, Houston, TX; Baylor College of Medicine, Houston, TX; Baylor College of Medicine, Houston, TX; Baylor College of Medicine, Houston, TX; Baylor College of Medicine, Houston, TX; Baylor College of Medicine, Houston, TX; Baylor College of Medicine, Houston, TX

## Abstract

**Background:**

Cardiovascular implantable electronic device (CIED) associated infection is the most common indication for lead extraction. While the need for complete device removal is well established, this study is the first to examine the impact of antimicrobial duration on clinical outcomes.

**Figure 1:**
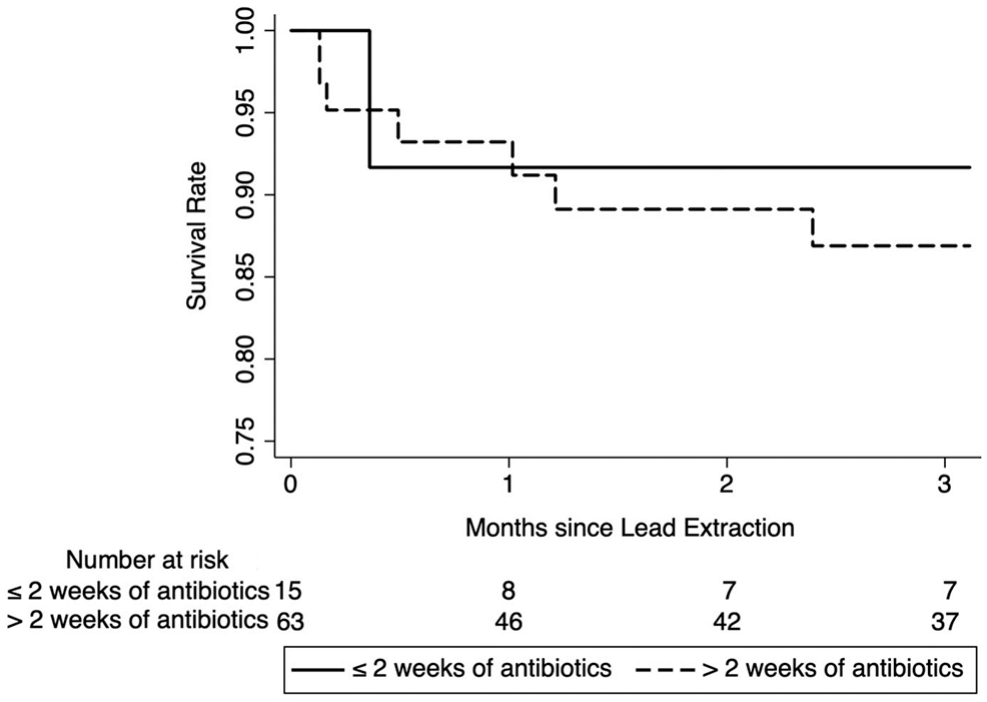
Overall Survival Post-CIED Lead Extraction Stratified by Duration of Antibiotic Therapy

**Methods:**

We reviewed all patients who underwent CIED lead extraction at our institution between June 2013 and December 2023. Patients who had extraction for a primary indication of bacteremia or lead-associated vegetation were included. Duration of antibiotics prescribed was stratified by initial decision to treat, ≤ 2 weeks or > 2 weeks post-extraction. Measured outcomes were all-cause mortality at 90 days, rates of recurrence or relapse of bacteremia from the same organism, infectious complications, and post-operative disposition to intensive care unit (ICU).

**Results:**

Of 747 patients reviewed, 79 cases met inclusion criteria. Baseline characteristics were very similar between cohorts. Median duration of antibiotic therapy was 12.6 and 38.6 days, respectively. Kaplan-Meier survival analysis showed no significant difference in survival (p= 0.438, HR 0.693, 95% CI 0.085-5.652). There was no difference in rates of recurrent bacteremia (7% vs 6%, p=0.952), infectious complications (27% vs 30%, p=0.817), hospital length of stay (mean 9.9 vs 13.3, p=0.360), post-operative ICU disposition (0% vs 17%, p=0.112), and rates of cardiac arrest (7% vs 5%, p=0.577) between patients who were prescribed ≤ 2 weeks of antibiotics versus > 2 weeks of antibiotics, respectively. Relapse or recurrence was seen in 5 patients, all of whom had *Staphylococcus aureus* (n=3) or *Serratia* sp. (n=2) and either an LVAD or valve replacement.

**Conclusion:**

Shorter durations (≤ 2 weeks) of antimicrobial therapy after CEID lead extraction was not associated with increased mortality or higher rate of recurrent bacteremia. Among the few patients that experienced relapse or recurrence in both groups, all were associated with high-risk organisms for secondary seeding of other cardiovascular prostheses. Larger studies are needed to define optimal antibiotic duration and determine risk factors for recurrent bacteremia in this population.

**Disclosures:**

All Authors: No reported disclosures

